# Serum supplementation during in vitro fertilization of sheep oocytes influences blastocyst quality through the differential abundance of mRNA transcripts

**DOI:** 10.1111/rda.14161

**Published:** 2022-05-31

**Authors:** Irene Sánchez‐Ajofrín, Patricia Peris‐Frau, Olga García‐Álvarez, María del Rocío Fernández‐Santos, Vidal Montoro, José Julián Garde, Ana Josefa Soler

**Affiliations:** ^1^ Instituto Regional de Investigación Científica Aplicada (IRICA) UCLM Ciudad Real Spain; ^2^ SaBio IREC (CSIC‐UCLM‐JCCM) ETSIAM Albacete Spain; ^3^ Department Animal Reproduction (INIA‐CSIC) Madrid Spain

**Keywords:** embryo quality, gene expression, in vitro fertilization, serum, sheep

## Abstract

Incubation with estrous sheep serum (ESS) is required to induce in vitro capacitation of spermatozoa during in vitro fertilization of small ruminants. However, the effect of adding different serum concentrations in the fertilization media on the quality of resulting blastocysts has not yet been studied. Here, 298 sheep oocytes were co‐incubated with capacitated spermatozoa with either 10% or 2% ESS. There were no differences between treatments in cleavage (10% ESS: 63.81 ± 5.87% and 2% ESS: 45.31 ± 5.87%) and blastocyst rates (10% ESS: 20.83 ± 2.12% and 2% ESS: 15.93 ± 2.12%). Nonetheless, in vitro‐produced blastocysts from the 10% ESS treatment showed a higher transcript abundance of mRNAs involved in apoptosis (*ITM2B* and *BCL2*), antioxidant defence (*GPX1*) and growth‐related imprinting (*IGF2R*). Our data suggest that ESS supplementation during in vitro fertilization can influence the quality of sheep embryos at later stages of development by increasing the transcription of developmentally important genes.

## 1 INTRODUCTION

Despite its undefined nature, serum is routinely used to supplement culture media as a source of growth factors, proteins and antioxidants for promoting embryo development in vitro (Lim et al., 2007).

The most common fertilization medium for sheep oocytes is synthetic oviductal fluid (SOF) supplemented with estrous sheep serum (ESS) since ram spermatozoa need ESS for successful in vitro capacitation (García‐Álvarez et al., 2015). Nevertheless, serum may cause alterations in the embryo gene expression and cryotolerance due to increased intracellular lipid droplet accumulation (Rizos et al., 2003; Sudano et al., 2011) and developmental anomalies such as foetal overgrowth (Young et al., 2001).

Numerous studies in small ruminant species have revealed that adding 2% serum in the fertilization medium results in higher blastocyst rates than those cultured with 20% serum (Reviewed by Zhu et al., 2018). However, there is no information regarding its effect on the quality of resulting blastocysts.

With this background, we aimed at evaluating the impact of ESS during sheep in vitro fertilization phase on subsequent embryo production and the mRNA transcription of developmentally important genes in blastocysts.

## 2 MATERIALS AND METHODS

All the chemicals were purchased from Merck Life Sciences.

### 2.1 Oocyte collection and in vitro embryo production

Adult sheep ovaries from Manchega or mixed breed between 2 and 8 years old were collected at a local abattoir and transported at 30°C in physiological saline (8.9 g/L) supplemented with penicillin (0.1 g/L). Immature cumulus–oocyte complexes were isolated and cultured in maturation medium: TCM199 with gentamycin (4 μl/ml), cysteamine (100 μM), FSH and LH (both 10 μg/ml) and 10% FCS at 38.5°C and 5% CO_2_.

After 22 hr, groups of 40–45 mature oocytes were placed in two different fertilization media: SOF + 10% ESS or SOF + 2% ESS. The Germplasm Bank of UCLM provided sperm samples. Frozen‐thawed spermatozoa from two Manchega breed rams were selected by centrifugation on a Percoll® gradient (45/90%), capacitated in SOF + 10% ESS and co‐incubated with oocytes at a final concentration of 10^6^ spermatozoa/ml at 38.5°C under 5% CO_2_, 5% O_2_ and 90% N_2_.

After 18 hr post‐insemination, presumptive zygotes were cultured in droplets of 20–25 μl of SOF and 3 mg/ml BSA for 8 days at 38.5°C and a humidified atmosphere of 5% CO_2_, 5% O_2_ and 90% N_2_. Cleavage rate was assessed at 48 hr post‐insemination, and blastocyst yield was recorded on days 6, 7 and 8. All expanded blastocysts were snap‐frozen and stored at −80°C for mRNA analysis.

### 2.2 mRNA transcript analysis

The relative quantification of mRNA transcript abundances was measured by qPCR as previously described (Sánchez‐Ajofrín et al., 2020) in three pools of ~10 blastocysts each (10% ESS: pools of 10, 10 and 11 blastocysts; 2% ESS: pools of eight blastocysts each) randomly selected from three replicates. Briefly, total RNA from 55 blastocysts was isolated using a magnetic bead‐based extraction (Dynabeads® Kit; Invitrogen). The qPCR (preincubation of 50°C for 2 min and 95°C for 2 min followed by 40 cycles of 95°C for 15 s and 60°C for 1 min) was performed with 10 μl PowerUp™ SYBR® Green Master Mix (Applied Biosystems), 2 μl cDNA and 400 nM of each primer (Table **1**). Target gene expression was normalized against that of the endogenous control *H2AFZ*.

**TABLE 1:** Primer information

Gene	Primer sequence (5′–3′)		Size (bp)
	Forward	Reverse	
*H2AFZ*	ATTGCTGGTGGTGGTGTCAT	ACTGGAATCACCAACACTGGA	147
*SOD2*	GCTTACAGATTGCTGCTTGT	AAGGTAATAAGCATGCTCCC	101
*GPX1*	GCAACCAGTTTGGGCATCA	CTCGCACTTTTCGAAGAGCATA	116
*SCH1*	GTGAGGTCTGGGCAGAAGC	GGTTCGGACAAAAGGATCACC	335
*TP53*	GACTCTCGTGGTAACCTGCT	AATTTTCTTCCTCAGTGCGGC	91
*ITM2B*	GTCCCAGAGTTTGCAGATAGTGA	GGAATCACATAGCACTTATCCAGGTT	104
*BAX*	GTTGTCGCCCTTTTCTACTTTGC	CAGCCCATGATGGTCCTGATC	89
*BCL2*	GGAGCTGGTGGTTGACTTTC	CTAGGTGGTCATTCAGGTAAG	518
*NRF1*	CTGTCGCCCAAGTGAATTATTCG	TGTAACGTGGCCCAGTTTTGT	67
*POLG2*	CTTCTGGGAAACTACGGGAGAAC	GTAGCCTCTTGTTTACCAGATCCA	84
*AKR1B1*	CGTGATCCCCAAGTCAGTGA	AATCCCTGTGGGAGGCACA	152
*IGF2R*	GCTGCGGTGTGCCAAGTGAAAAAG	AGCCCCTCTGCCGTTGTTACCT	201
*GJA1*	TGCCTTTCGTTGTAACACTCA	AGAACACATGAGCCAGGTACA	143

### 2.3 Statistical analysis

The IBM SPSS 24 software was used to carry out statistical analysis. Data were tested for normal distribution (Kolmogorov–Smirnov and Shapiro–Wilk tests). The effect of ESS concentration on embryo production and blastocyst quality was studied by factorial ANOVA followed by the Bonferroni post hoc test. Replicates (seven for embryo production and three for embryo quality) were considered in this model. A *p‐*value ≤.05 was considered statistically significant. Results are presented as mean ± SEM.

## 3 RESULTS

**FIGURE 1:**
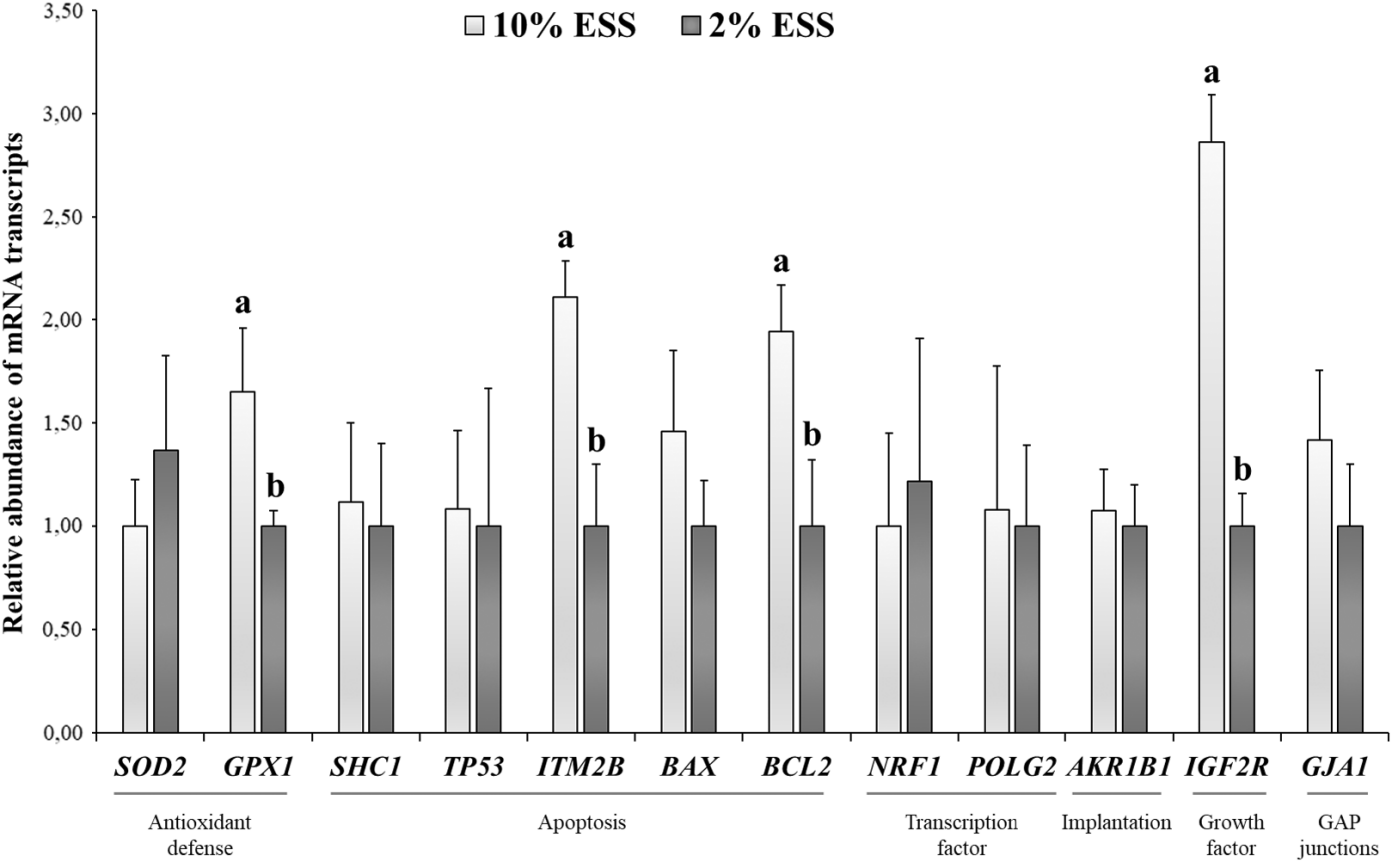
Relative abundance of mRNA transcripts in sheep in vitro‐produced blastocysts after supplementation of fertilization media with 10% ESS and 2% ESS. Results are expressed as means ± SEM. ^a,b^Different letters indicate significant differences (*p* ≤ .05)

There were no significant differences between treatments on cleavage and blastocyst rates (*p* > .05; Table 2). However, the supplementation of 10% ESS in the fertilization media showed a significantly (*p* ≤ .05) higher abundance of mRNA transcripts related to apoptosis (*ITM2B* and *BCL2*), antioxidant defence (*GPX1*) and imprinting (*IGF2R*; Figure 1) on in vitro‐produced blastocysts.

**TABLE 2:** Effect of concentration of ESS supplementation during fertilization on embryo

Treatment	*n*	Cleavage (%)	Expanded blastocyst (%)	
			Total	Cleaved
10% ESS	150	63.81 ± 5.87	20.83 ± 2.12	33.78 ± 5.80
2% ESS	148	45.31 ± 5.87	15.93 ± 2.12	29.11 ± 5.80

Data are mean ± SEM.

## 4 DISCUSSION

In this study, we found that the presence of serum (10% ESS) during fertilization of sheep oocytes influences the quality of developing blastocysts by increasing the transcription of mRNAs involved in apoptosis, antioxidant defence and growth‐related imprinting. To the best of our knowledge, this is the first study that demonstrates that ESS supplementation during sperm and oocyte co‐incubation can directly affect the quality of the embryo at a later developmental stage.

Although the addition of 10% and 2% ESS produced similar embryo rates, it is noteworthy to say that the greatest values were obtained with 10% ESS treatment in all parameters. Parallel to these results, López‐Saucedo et al. (2014) reported that 20 and 2% ESS concentrations during fertilization yielded similar embryo rates in goats.

Serum contains beneficial substances for embryonic development such as growth factors, antioxidants, amino acids and heavy metal chelating agents. Despite the fact that adding growth factors to standard IVF systems is currently controversial, serum has and still is widely used in the fertilization culture media in sheep (Zhu et al., 2018). In addition, the presence of serum in the embryo culture medium can affect the quality of resulting blastocysts as analysed by qPCR and embryo cryotolerance methods (Rizos et al., 2003). As widely known, differentially expressed mRNA transcripts is an important parameter to consider in the assessment of embryo quality (Cánepa et al., 2014), and the mRNA transcripts used in the present study are developmentally important gene transcripts that have been previously linked to the quality of blastocysts in the ruminant species (Bermejo‐Álvarez et al., 2010; Cañón‐Beltrán et al., 2021; González et al., 2021; Warzych et al., 2007).

The alteration of *IGF2R* we observed agrees with growing evidence indicating that serum can interfere with the expression of growth‐related imprinting genes in blastocysts (Fernández‐Gonzalez et al., 2004). *IGF2R* belongs to a group of genes subjected to imprinting, which involves DNA remodelling mechanisms such as methylation. IGF2R is an ‘elimination receptor’ – it does not activate any intracellular signalling pathway but works locating ligand IGF2 in excess for its lysosomal destruction. Human and bovine serum contains IGF2, and down‐regulation of *IGF2R* in sheep embryos cultured with serum is associated with an increase in IGF2, leading to foetal overgrowth (Young et al., 2001). Interestingly, we observed a greater abundance of *IGF2R* in embryos fertilized with 10% ESS compared with 2%. In our study, oocytes and early zygotes were only exposed to ESS for 18 hr, while in the previously mentioned study, embryos were kept in culture for an extended period. It is possible that, even at high doses, using serum at earlier stages of in vitro development or for shorter times may overcome the problems associated with the alteration of imprinting genes.

We found that the relative abundance of antioxidant‐related *GPX1*, anti‐apoptotic *BCL2* and pro‐apoptotic *ITM2B* mRNA was greater in 10% ESS‐derived blastocysts. *GPX1* protects against oxidative damage, promoting cell survival under stressed conditions. *ITM2B* stimulates apoptosis by losing mitochondrial membrane potential and permeability, cytochrome c release and caspase activation. Yet, the overexpression of *BCL2* can block *ITM2B* action by inhibiting the apoptotic mitochondrial pathway (Fleischer et al., 2002).

In conclusion, notwithstanding the use of different ESS concentrations during fertilization did not influence the developmental capacity of sheep oocytes, the quality of resulting blastocysts was significantly affected since a 10% ESS serum concentration up‐regulated the transcript levels of several developmentally important genes. It will be interesting to see the effect these treatments may have in species in which serum is not commonly used in the fertilization media.

## 5 AUTHOR CONTRIBUTIONS

IS‐A performed the experiments, analysed the data and drafted the manuscript. PP‐F, OG‐A, MRF‐S and V‐M performed the experiments. JJG reviewed the manuscript. AJS designed the study, analysed the data and drafted the manuscript interest.

## 7 CONFLICT OF INTEREST

The authors declare no conflict of interest.

8

## Data Availability

The data that support the findings of this study are available from the corresponding author upon reasonable request.
